# Anaplastic large cell lymphoma ALK-positive variant of primary bone lymphoma associated with melanoma

**DOI:** 10.1093/omcr/omad025

**Published:** 2023-03-25

**Authors:** Victoria V Tutaeva, Maria R Ovsiannikova, Alexander N Bobin, Alexey V Gorbunov, Sergey I Kurbanov, Oleg A Rukavitsin

**Affiliations:** Department of Hematology, The Main Military Clinical Hospital Named After N.N. Burdenko, Moscow 105094, Russia; Department of Pathology, The Main Military Clinical Hospital Named After N.N. Burdenko, Moscow 105094, Russia; Department of Pathology, The Main Military Clinical Hospital Named After N.N. Burdenko, Moscow 105094, Russia; Department of Radiology, The Main Military Clinical Hospital Named After N.N. Burdenko, Moscow 105094, Russia; Department of Radiology, The Main Military Clinical Hospital Named After N.N. Burdenko, Moscow 105094, Russia; Department of Hematology, The Main Military Clinical Hospital Named After N.N. Burdenko, Moscow 105094, Russia

## Abstract

We describe in detail a case of the anaplastic large cell lymphoma (ALCL) type of primary bone lymphoma, which initially was diagnosed and treated as osteomyelitis. The diagnosis was delayed because of unspecific clinical symptoms and uncertain radiographs and histology. Only relapse of the lymphoma from the same area with involvement of the soft tissue and local lymph nodes allowed to establish a correct diagnosis and start treatment. Also, in this case, we observed the development of the second cancer (melanoma), which has the same cytogenetic abnormality as ALCL (t[2;5]).

## INTRODUCTION

Primary bone lymphoma (PBL) is a rare type of extranodal lymphoma, representing 3% of all malignant bone tumors and 1% of all malignant lymphomas. The etiology of PBL is unknown; viral agents and immunosuppression are implicated in some cases. Cytogenetic and molecular abnormalities are involved in the pathophysiology of different types of lymphomas.

Most cases (70–80%) of PBL are diffuse large B-cell lymphomas. The anaplastic large cell lymphomas (ALCL) type of PBL is extremely rare (1%), and only 22 cases were described by 2018 in the English-language literature [1].

The prognosis of ALCL that primarily involves bone is unfavorable compared with general PBL. Clinical symptoms in patients with PBL are unspecific; the differential diagnosis usually requires much time, leading to delays in treatment [2]. This fact has profound implications for the disease prognosis. Our case demonstrates the difficulty in obtaining the correct diagnosis of a rare form of PBL, ALCL anaplastic lymphoma kinase (ALK)-positive (ALK+ ALCL). In addition, we highlight a cytogenetic anomaly, t (2;5), as a common factor in the development of lymphoma and melanoma—the second neoplasm of the patient.

## CASE REPORT

**Figure 1:**
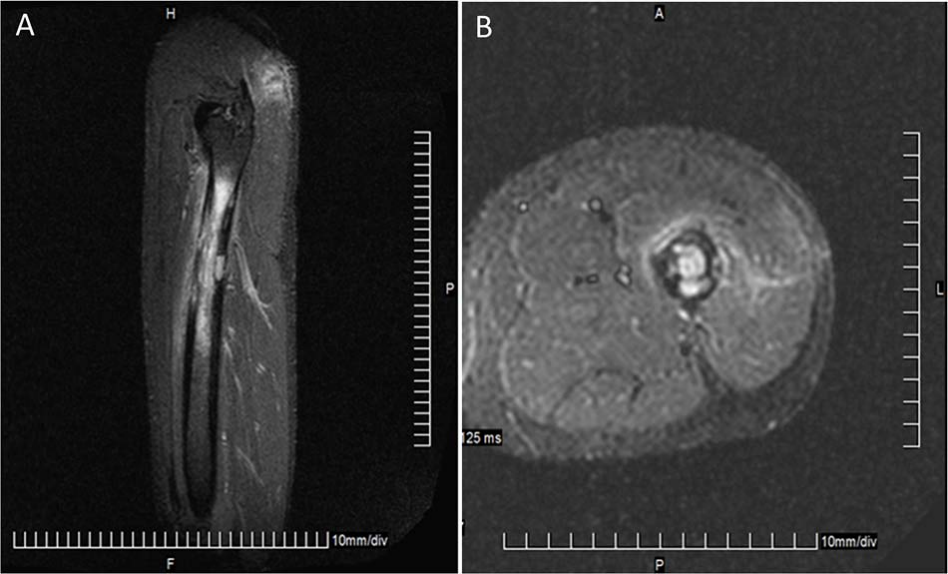
Coronal image (**A**) and transversal image (**B**) showing irregular lytic lesions involving cortical destruction of the proximal right femur.

**Figure 2:**
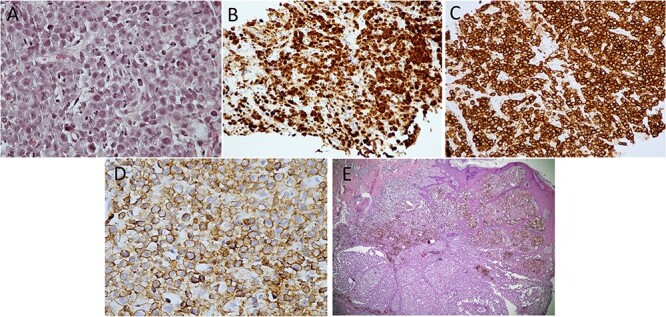
Immunohistochemistry of PBL. (**A**) Hematoxylin–eosin staining (×400 magnification). (**B**) ALK staining (×100 magnification). (**C**) CD30 staining (×100 magnification). (**D**) CD45 staining (×100 magnification). (**E**) Ulcerated melanoma hematoxylin–eosin staining (×40 magnification).

A 35-year-old Caucasian man began to have spontaneous pain in the left hip in August 2017. He used nonsteroidal anti-inflammatory drugs, which relieved the pain. He was admitted to the hospital in September 2017 complaining of intense pain that worsened when he did weight-bearing activities. He also reported that pain became more intense over the last month and sometimes he had it at rest. The physical examination of the left hip and left hip joint was unremarkable. Laboratory examinations revealed a leucocyte count of 4.1 × 10^3^/μl (the normal range: 4.5–11 × 10^3^/μl), C-reactive protein concentration of 2.6 mg/dl (<0.9 mg/dl) and erythrocyte sedimentation rate of 18 mm/h (0–22 mm/h), and screening for infectious organisms, including microscopy, polymerase chain reaction and culture for *Mycobacterium tuberculosis* was negative. A bone scan was performed. Because of the morphology on the magnetic resonance imaging (MRI), osteomyelitis of the left proximal femur was suspected (Fig. 1). An open biopsy of the left femur was performed. Histology revealed normal tissue, no signs of osteomyelitis were found. The patient consulted with a hematologist/oncologist and surgeon. Based on the data, physical exam and MRI data, a diagnosis of chronic osteomyelitis of the left proximal femur with unknown etiology was made. Bone necrotomy was performed in January 2018. Samples of blood and bone fragments were taken from the damaged area for microbiological analysis. After 5 and 7 days, the samples analyzed showed no bacterial or fungal growth, and the patient received broad-spectrum antibiotic therapy. The operative wound healed without any complications. The patient’s condition improved, with no pain or other complaints. Twelve months later (January 2019), the patient was presented to the main military hospital with gradually progressive pain and swelling of the left hip. Positron emission tomography/computed tomography (PET/CT) scan imaging demonstrated tense metabolic activity in the proximal part of the left femur and soft tissue lump formation (164 × 114 × 200 mm in size and maximum standardized uptake value of 33.9). Local lymph nodes were involved: left internal iliac nodes (15 × 8 mm) and left inguinal nodes (15 × 9 mm). Next, a biopsy of the tumor tissue of the left hip was performed. Histological examination showed large, atypical cells with abundant eosinophilic cytoplasm and eccentric horseshoe nuclei. Immunohistochemical stains were positive for CD45, CD30 and ALK-1 but negative for CD20, CD3 and CDPAX5. Ki-67 showed a very high nuclear proliferative index with positive staining in 80–90% of the cells (Fig. 2A and D). Fluorescent in situ hybridization showed cytogenetic translocation—t(2;5). A final diagnosis of PBL—ALK-positive ALCL—was made. The patient started chemotherapy with the cyclophosphamide, doxorubicin, vincristine, etoposide and prednisone regimen for six cycles. A PET/CT scan was done at the end of chemotherapy to assess the response and consider further management. The PET scan showed no significant metabolically active disease in the primary bone lesion site or anywhere else in the body. The patient was given local radiotherapy with a dose of 30 Gy and was instructed to follow-up 12 months post-chemotherapy. There is no evidence of recurrence to date. However, 6 months later, the patient was admitted to the hospital complaining of a progressively enlarged dark pigmented lesion (10 mm) with bleeding and ulceration on his lower back. An excision biopsy of the lesion was performed. Pathology showed an ulcerated malignant melanoma (Fig. 2E). Thus, based on Warren and Gates criteria, we diagnosed double primary malignancies. The patient was referred to an oncologist for further treatment. The patient has been treated by nivolumab for 12 month. Currently he is in complete remission for both cancers.

## DISCUSSION

PBL is defined as (i) a single bone lesion, with or without the involvement of regional lymph nodes; or (ii) multiple bone lesions without lymph nodal or visceral disease. The differential diagnosis generally considers chronic osteomyelitis, primary bone sarcoma, Ewing sarcoma, rhabdomyosarcoma, small cell lung carcinoma and small cell osteosarcoma [2, 3].

Diagnostic workup is complex and includes different types of radiology exams and histopathology tests. In most cases, radiographic imaging of the affected area is performed, but radiographic findings for patients with PBL make it challenging to distinguish it from other primary bone tumors. PET/CT and MRI are much more sensitive and effective methods in the case of PBL. Biopsy of the lesions is another critical investigation for diagnosis; however, according to the literature [4], a bone biopsy is not informative for a significant fraction of patients with PBL. Also, PBL systemic symptoms are usually absent in most cases, and no clinical signs of malignant lymphoma exist. The combination of these facts explains why PBL is often misdiagnosed, and the start of treatment is delayed in patients with PBL, which can have severe effects on the prognosis of the disease.

In our case, the MRI and bone biopsy did not provide clues to the correct diagnosis.

Presumably, the surgical resection of the damaged bone led to a significant reduction of the tumor mass and, consequently, significantly delayed relapse of the disease (⁓12 months).

Relapse of the lymphoma at the area of primary femur damage allowed to perform soft tissue biopsy and established the ALCL diagnosis.

Retrospective analysis of the case re-evaluated osteomyelitis diagnosis, and the final diagnosis of PBL ALCL was made. A complete remission was achieved after chemotherapy. A follow-up investigation after 18 months confirmed metabolic remission of PBL; however, the primary melanoma was diagnosed.

Molecular characterization of melanoma revealed BRAF and ALK mutation.

Several translocations within the ALK locus (⁓22) are implicated in the pathogenesis of several cancer types. Among the 30–60% of systemic ALCL cases positive for ALK, 70–80% express the t (2;5) translocation [5]. Another cancer that is positive for ALK is melanoma. ALK translocation was first identified in patients with acral melanoma in Southern China [6, 7]. A novel isoform of ALK^ATI^ has been described in primary and metastatic melanomas. Both ALK^ATI^ and ALK rearrangements led to an increase of the ALK expression, which is an oncogenic driver [8]. Janostiak *et al*. have shown that ALK is a driver of acquired BRAF kinase inhibitor (BRAFi) resistance [9]. The ALK inhibitor ceritinib inhibited BRAFi-resistant melanoma in cell culture and mice [9]. Thus, additional investigations are required to reveal the clinical significance of ALK alterations in melanoma.

In the reported case, the ALK mutation is highly likely to have played an important role in the pathogenesis of both diseases: PBL ALCL ALK+ and melanoma. Further investigation of ALK mutations and individualized, targeted therapy will allow the treatment of patients with different neoplasms carrying common genome abnormalities.
